# A brief comparison of polygenic risk scores and Mendelian randomisation

**DOI:** 10.1186/s12920-023-01769-4

**Published:** 2024-01-02

**Authors:** Victoria Garfield, Emma L. Anderson

**Affiliations:** 1 MRC Unit for Lifelong Health and Ageing, Institute of Cardiovascular Science, UCL, 1-19 Torrington Place, London, WC1E 7HB UK; 2 Division of Psychiatry, University College London, 149 Maple House, Tottenham Court Road, London, W1T 7NF UK

**Keywords:** Mendelian randomisation, Polygenic risk scores, Genome-wide association studies, Horizontal pleiotropy

## Abstract

Mendelian randomisation and polygenic risk score analysis have become increasingly popular in the last decade due to the advent of large-scale genome-wide association studies. Each approach has valuable applications, some of which are overlapping, yet there are important differences which we describe here.

## Main text

There is often misconception surrounding the differences and similarities between polygenic risk score analysis and Mendelian randomisation, and when one method should be applied over the other. In this article we briefly describe what polygenic risk scores (PRS) and Mendelian randomisation are, their respective strengths and limitations, whether PRS and Mendelian randomisation (MR) are equivalent, and as such, whether we can use these methods interchangeably.

## What are polygenic risk scores (PRS)?

Polygenic risk scores (sometimes also referred to as genetic risk scores) estimate an individual’s genetic predisposition to a trait (e.g., LDL-cholesterol) or disease (e.g., type-2 diabetes) [1]. A PRS is usually calculated using individual-level genotypes and data from genome-wide association studies (GWAS). An unweighted PRS simply reflects the sum of an individual’s risk alleles. Unweighted PRS do not take into account the relative magnitude of effect of each genetic variant on the trait of interest. Weighted PRSs are the sum of an individual’s risk alleles, weighted by the effect sizesreported in published GWAS (e.g., log(beta) or beta coefficient). The number of variants to include in a PRS depends on the intended application, whether it is to assess causality or prediction, as more variants is better for the latter, but this increases the chances of including pleiotropic variants. Table 1 outlines some of the potential applications of PRS. For more extensive details on PRS methods, see [1, 2].

**Table 1:** A non-exhaustive list of potential applications of PRS and MR

Applications	Polygenic risk score analysis	Mendelian Randomisation	Potential for bias due to horizontal pleiotropy?	Requires individual level data?^a^
Identifying an effect of an exposure on an outcome	✓	✓	✓	MR = NO PRS = YES
Comparing outcomes in high versus low genetic risk for an exposure	✓	X	✓	YES
Examining gene x environment interactions	✓	✓	✓	YES^b^
Prediction modelling	✓	X	X	YES
Quantifying shared genetic aetiology	✓	X	X	NO
Identifying downstream effects of liability to a disease	✓	✓ (“reverse MR” [3])	✓	MR = NO PRS = YES
Identifying biomarkers of disease	✓	✓	✓	MR = NO PRS = YES

^a^Where this column is marked ‘NO’, this indicates that the research question can likely be addressed using summary level data (from GWAS) instead

^b^For identifying gene environment interactions using MR, this can in theory be conducted with summary data provided that both the exposure and the outcome GWASs have been performed within the subgroups of interest. For example, if we examine the effect of BMI on dementia risk in APOE4 versus APOE3 carriers, we would need the GWAS of BMI and the GWAS of dementia to be performed separately in APOE4 carriers and APOE3 carriers. However, in practise, most summary level data for both our exposure and outcome of interest are not available within subgroups, so we usually require individual level data to examine gene x environment interactions

## What is Mendelian randomisation?

Mendelian randomisation uses SNPs (i.e., single nucleotide polymorphisms – SNPS –) or common genetic variants as instrumental variables (IVs) for an exposure of interest, rather than using the observed phenotype, to examine whether the exposure (or liability to an exposure if it is binary) has an effect on an outcome of interest [4]. MR exploits the unique properties of common genetic variants and the fact that genes are randomly allocated from parents to offspring during gamete formation [5]. As such, MR exploits Mendel’s laws of ‘Independent Assortment’ and ‘Segregation’. In practice, an MR has three assumptions that need to be upheld for it to be valid: 1) robustness of association between SNPs and the exposure to be instrumented, 2) no association (horizontal pleiotropy) between the SNPs for the exposure and the outcome that does not go via the exposure (Table 1), and 3) the SNP-outcome relationship is unconfounded. MR can be performed in both individual-level and summary-level (i.e. genome-wide association study summary statistics) data settings [6], which each have different advantages and disadvantages, summarised in Lawlor et al. [6].

## PRS vs. MR: understanding their similarities and differences using an applied example: body mass index (BMI) and sleep duration

**Fig. 1:**
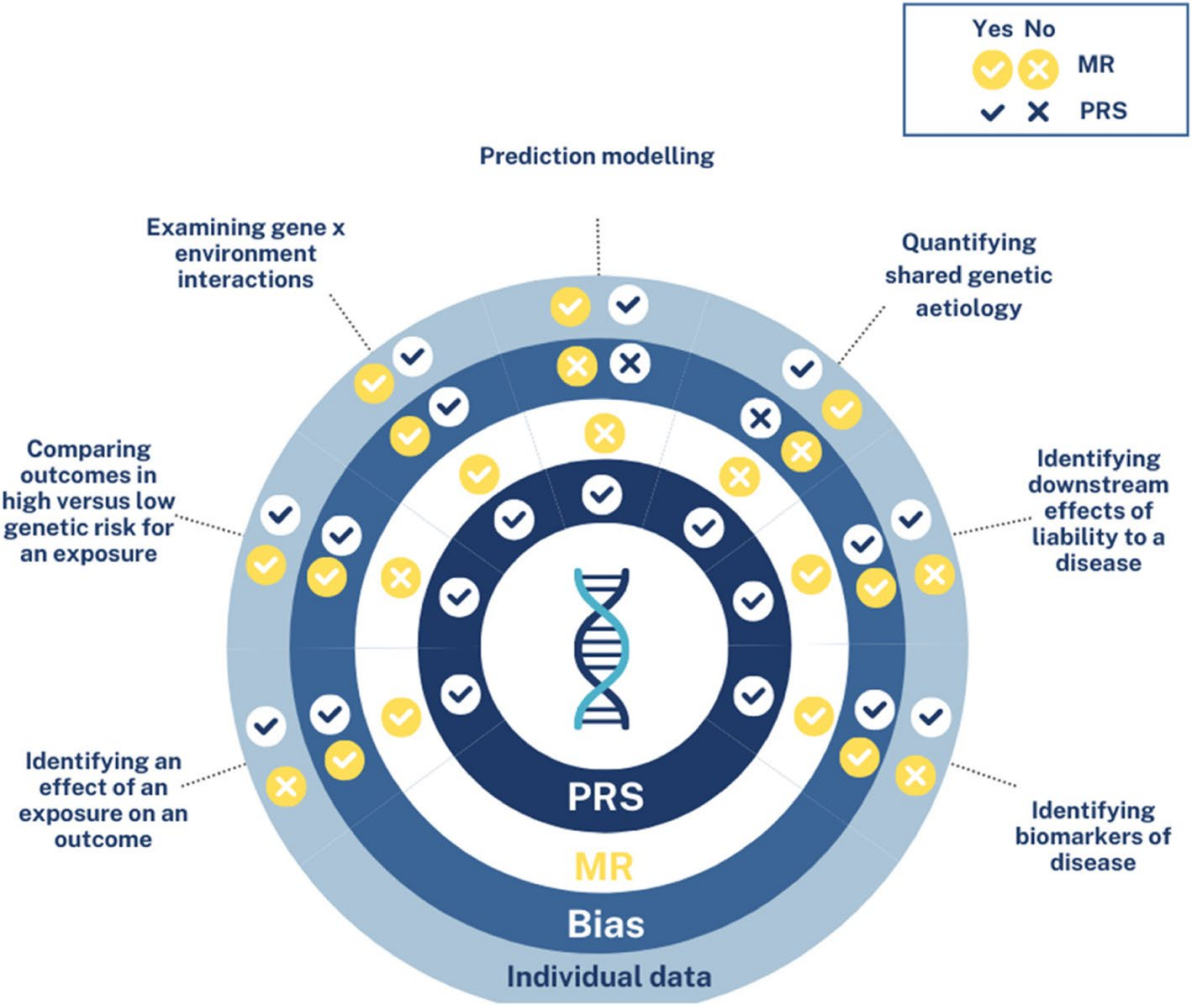
Graphical representation of the conceptual similarities and differences between PRS and MR

The relationship between BMI and sleep duration has been extensively investigated via epidemiological and experimental studies [7]. The first PRS study which aimed to investigate *shared genetic aetiology* between BMI and (self-reported) sleep duration was published in 2019 [8]. Then, a comprehensive and well powered MR study of BMI and sleep duration emerged earlier this year [9], which investigated *causality* between this exposure and outcome. The two studies had distinct objectives and thus, employed different approaches (e.g., the PRS study employed nine different PRS with varying numbers of SNPs, whereas the MR study used a genome-wide significant 67-SNP instrument). However, both studies reached similar conclusions, and the analyses produced comparable results, such that there was little shared genetic aetiology, and no evidence of a causal relationship between BMI and self-reported sleep duration in adults. Table 2 presents a detailed account of similarities and differences between PRS and MR, while Fig. 1 is a graphical representation of the conceptual similarities and differences between the two methods.

**Table 2:** Similarities vs. differences between Mendelian randomisation and polygenic risk score approaches

*Similarities*
• Both MR and PRS exploit results from GWAS (summary statistics) • Both can be performed using individual- or summary-level data, however most applications of PRS apart from estimating shared genetic aetiology requires individual level data • Both MR and PRS can be used to estimate an effect of liability to an exposure on an outcome • PRSs can be utilised within in a one-sample MR framework • Provided heterogeneity is low, and the PRS is scaled to the exposure, MR and PRS should give approximately the same answer (see examples of PRS and MR studies of BMI-sleep duration below) • Both MR and PRS rely on the R^2^ (variance explained) as a metric of total strength of the instrument [10]
*Differences*
• PRS and one-sample MR combine all SNPs into a score, whereas summary level MR is done on a per SNP basis and meta-analysed • Methods for examining and correcting for bias due to horizontal pleiotropy are better developed for MR than for PRS. It is not possible to formally detect and correct for pleiotropy using PRS, but for some PRS applications, horizontal pleiotropy does not cause bias (Table 1) • For a given sample size, PRS have greater power than MR; thus PRS are often useful for smaller samples. However, summary level MR usually have much larger sample sizes as a result of using large GWAS • PRS are generally more flexible in their applications than MR (Table 1) • Less likely to suffer weak instrument bias [11] with a PRS as alleles aggregated into a score and thus usually explains more variance in the exposure. This relates to average strength of the instrument, which is estimated using the F-statistic (should be > 10 for an instrument of good average strength) [11]

## Conclusions

PRS and MR both have useful applications in aetiological epidemiology. PRS are useful in the case of weak genetic instruments or smaller sample sizes, as aggregation of alleles into a score increases the variance explained in the exposure, and thus increases power. MR is useful for larger sample sizes and can also be performed on publicly available summary data. MR is the preferred method for identifying and correcting for potential bias due to horizontal pleiotropy as methods are more widely developed. PRS are typically more flexible in their potential applications.

## Data Availability

Not applicable.

## Abbreviations

BMI: Body mass index
GWAS: Genome-wide association study
IV: Instrumental variable
MR: Mendelian randomisation
PRS: Polygenic risk score
SNP: Single nucleotide polymorphism
